# Successful treatment of refractory status asthmaticus with omalizumab: a case report

**DOI:** 10.1186/s13223-021-00629-z

**Published:** 2021-12-09

**Authors:** Jan Benes, Roman Skulec, Dalibor Jilek, Ondrej Fibigr, Vladimir Cerny

**Affiliations:** 1 Faculty of Medicine in Hradec Kralove, Charles University in Prague, University Hospital Hradec Kralove, Hradec Kralove, Czechia; 2 Dept. of Anesthesiology, Perioperative Medicine and Intensive Care, Masaryk Hospital, J.E. Purkinje University, Usti nad Labem, Czechia; 3 Faculty of Health Studies, J.E. Purkinje University, Usti nad Labem, Czechia; 4 Dept. of Anesthesiology and Intensive Care, Faculty of Medicine in Hradec Kralove, Charles University in Prague, University Hospital Hradec Kralove, Hradec Kralove, Czechia; 5 Center for Research and Development, Dept. of Anesthesiology and Intensive Care, Faculty of Medicine in Hradec Kralove, Charles University in Prague, Hradec Kralove, Czechia; 6 Dept. of Anesthesia, Pain Management and Perioperative Medicine, Dalhousie University, Halifax, Canada; 7 Emergency Medical Service of the Central Bohemian Region, Kladno, Czechia; 8 Department of Clinical Immunology and Allergology, Masaryk Hospital, J.E. Purkinje University, Usti nad Labem, Czechia; 9 Department of Pneumology, Third Faculty of Medicine, Charles University and University Hospital Bulovka, Prague, Czechia

## Abstract

Refractory status asthmaticus is the cause of rare cases of in-hospital death due to acute bronchial asthma. The most severe cases unresponsive to first, second and next line treatment may be fatal despite aggressive organ support with invasive ventilation and extracorporeal membrane oxygenation. Omalizumab, a humanized recombinant monoclonal anti-IgE antibody, is an approved add-on biological treatment for severe asthma. However, it is not indicated in an acute setting. Here, we report the case of a young patient with status asthmaticus fully dependent on extracorporeal membrane oxygenation refractory to any therapy for six days, who was successfully treated with omalizumab.

## Clinical implications statement

The presented case demonstrates the efficacy of omalizumab as a rescue therapy for refractory status asthmaticus associated with high IgE levels. Omalizumab should be considered in patients with status asthmaticus unresponsive to standard treatment.

## Background

Mortality due to bronchial asthma has gradually declined since the introduction of inhalational corticosteroids in the late 1980s, but it has plateaued since 2006 [1]. Refractory status asthmaticus is the cause of rare cases of in-hospital death due to acute bronchial asthma. Patients suffering from an asthma exacerbation may present with a variety of signs and symptoms. Dyspnea, chest tightness, cough and wheezing are common symptoms, but there is broad heterogeneity in the presentation of asthmatic patients. The features that characterize acute severe asthma are agitation, drowsiness or signs of confusion, significant breathlessness at rest, with the patient talking in words, tachypnea of more than 30 breaths per minute, use of accessory respiratory muscles, tachycardia of > 120 beats per minute, and pulsus paradoxus [2]. Chest radiographs are advised when the clinician needs to exclude conditions such as pneumonia, pneumothorax or atelectasis.

The pharmacological therapy of acute severe asthma should consist of a short acting beta agonist, ipratropium bromide, systemic corticosteroids and controlled oxygen therapy, and the clinician should consider the use of iv magnesium sulfate, high-dose inhaled corticosteroids, and β2 adrenergic receptor agonists such as epinephrine or terbutaline. Methylxanthines and leukotriene modulators may also be considered despite limited evidence for their efficacy. A mixture of helium (70–80%) and oxygen (20–30%) can be used for severe asthma exacerbations that are unresponsive to standard therapy or in patients with an upper airway obstruction component. A trial of non-invasive ventilation may be beneficial for a low-risk group of patients unresponsive to medical therapy [2]. Intubation and invasive mechanical ventilation are indicated if the respiratory failure is progressing and is unlikely to be reversed by further pharmacological therapy. Extracorporeal membrane oxygenation (ECMO) should be considered in patients who remain severely acidotic and hypercapnic despite conventional therapy. Here, we report the case of a patient with refractory status asthmaticus requiring extracorporeal membrane oxygenation, who was successfully treated with omalizumab. Omalizumab is a humanized recombinant monoclonal anti-IgE antibody that binds to free IgE in blood or membrane-bound IgE on B lymphocytes to form complexes, thus preventing IgE from interacting with mast cells and basophils. Administration of omalizumab leads to inhibition of allergen-induced inflammation, mainly by preventing mass cell degranulation and histamine release. Significant decrease of serum IgE also has long-term effects on pulmonary eosinophil infiltration and airway remodeling in asthma patients [3]. Several randomized control trials showed that omalizumab significantly reduces asthma exacerbations and improves lung function and symptoms in patients whose symptoms are inadequately controlled by inhaled corticosteroids and long-acting beta agonists [4–6]. However, omalizumab is not indicated in acute asthma. Omalizumab treatment is usually well tolerated and adverse effects are rare. The main adverse effect is anaphylaxis, with an incidence of 0.2%.

## Case report

A 25-year-old woman with a history of pollen allergy and bronchial asthma presented with severe shortness of breath preceded by several weeks of worsening symptoms after starting a new job in a textile warehouse with high exposure to dust. The patient had been inhaling ipratropium/fenoterol several times a day over the preceding few weeks and had undergone two courses of antibiotic treatment for suspected bacterial bronchitis. Despite worsening of the symptoms, the patient had not sought attention from a specialist and had not used inhaled corticosteroids or any other medication. The past medical history included atopic eczema and pollen allergy at preschool age, with documented strong polyvalent IgE sensitization and allergic asthma treated with medium dose inhaled corticosteroids and a long-acting beta agonist at school age. In adulthood, her asthma was well-controlled, with only occasional use of relief medication. The patient had no history of hospitalization due to asthma exacerbation. She had food allergies and oral allergic syndrome triggered by nuts, apples and tomatoes, and was an occasional smoker.

**Fig. 1.**
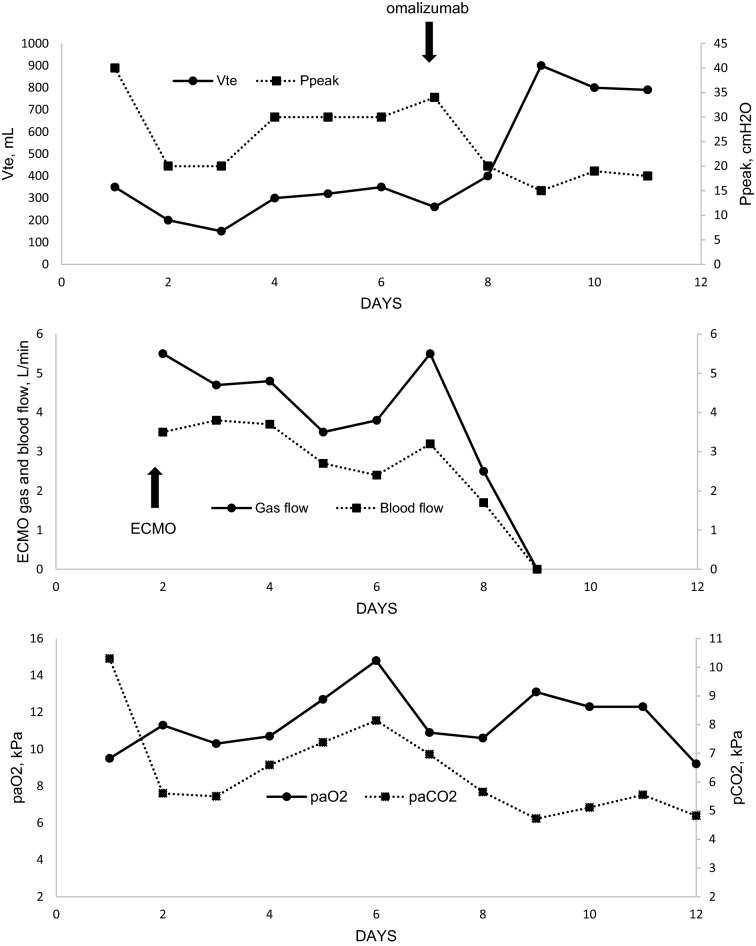
ECMO and ventilation parameters. Arrows show ECMO initiation on day 1 and omalizumab administration on day 7. Tidal volume and minute ventilation significantly improved within 24 h after omalizumab administration. ECMO was stopped on day 9 and the patient was extubated on day 11. ECMO, extracorporeal membrane oxygenation; P_peak_, peak inspiratory pressure; V_te_ expiratory tidal volume

Upon admission, the patient was tachypneic and complained of dry cough and breathlessness. Clinical examination revealed wheezing and tachypnea with prolonged expirium and hypoxemia, with S_P_O_2_ 88% on 5 L of oxygen. Chest X-ray was normal. Status asthmaticus was diagnosed, and standard treatment with nebulized salbutamol and intravenous methylprednisolone 1000 mg was initiated. The patient was admitted to the intensive care unit, and given nebulized ipratropium/fenoterol continuously, with 2 g intravenous magnesium sulfate initially, which was then adjusted to high normal plasma level. Terbutaline 2 mg/d and theophylline 720 mg/d (monitored by drug level) were added to the therapy. A trial of noninvasive ventilation was carried out, but it was not effective. Ten hours after admission, the patient was intubated due to exhaustion and mechanical ventilation was initiated. Terbutaline was replaced with intravenous adrenalin 10 μg repeatedly at short intervals as hemodynamically tolerated, which had only a moderate effect on ventilation. Regardless of intensive bronchodilator therapy, deep sedation, muscle paralysis and an aggressive ventilatory regimen, the condition of the patient was deteriorating into severe respiratory acidosis. Therefore, support with ECMO was initiated. The respiratory acidosis was rapidly corrected and p_a_CO2 normalized (Fig. 1). We continued treatment with methylprednisolone (1000 mg for 3 days, 60 mg from day 4), intravenous terbutaline, magnesium sulfate and theophylline. Oral montelukast 10 mg/day and intravenous bisuleptin 2 mg twice daily was added. Inhaled sevoflurane with an end-tidal concentration of 2.5% was added to ketamine (150 mg/h) and sufentanil (100 ug/h) sedation for its bronchodilatory effect. Ribavirin 600 mg twice daily, cefotaxime 2 g every 6 h and clarithromycin 500 mg twice daily were administered for 2 days until viral and bacterial lung infection was ruled out.

Echocardiogram and chest X-ray were normal, and microbiological examination of tracheal aspirate including viral and bacterial polymerase chain reaction tests were negative. Her total IgE level was hugely elevated, at 2087 kIU/L (reference range 0–150 kIU/L), with strong positivity for specific IgE against several inhaled allergens (Table 1). Her eosinophil count was normal, with an absolute value of 100/µL (reference range 0–500/µL).

**Table 1:** Pretreatment total and specific IgE levels

	Value	RAST^a^ class
Specific IgE: Aeroallergens		
Phadiatop (a mixture of common inhaled allergens)	92.10 PAU/L	5
tx9 tree pollens mix	96.20 kU/L	5
wx1 weed pollens mix	20.70 kU/L	4
gx3 grass pollens mix	94.90 kU/L	5
mx1 molds mix	7.70 kU/L	3
d1 *Dermatophagoides pteronyssinus*	8.36 kU/L	3
h1 house dust mix	62.10 kU/L	5
t215 r Bet v 1 PR-10 Birch	> 100.00 kU/L	6
t221 rBet v 2 Profilin, Birch rBet v 4 Birch	7.79 kU/L	3
g213 rPhl p 1, rPhl p 5b Timothy	68.10 kU/L	5
g214 rPhl p 7, rPhl p 12 Timothy	5.46 kU/L	3
Specific IgE: Food allergens		
fx5 foods mix	1.16 kU/L	2
f1 egg white	0.37 kU/L	1
f2 milk	0.29 kU/L	1
f3 fish (cod)	0.04 kU/L	0
f4 wheat	1.14 kU/L	2
f13 peanut	3.47 kU/L	2
f14 soybean	0.80 kU/L	1
f422 rAra h 1 peanut	0.02 kU/L	0
f423 rAra h 2 peanut	0.13 kU/L	0
f424 rAra h 3 peanut	0.11 kU/L	0

^a^Specific IgE assay classification with radioallergosorbent test (RAST) Class: 0: < 0.35 kU/L, 1: 0.35–0.69 kU/L, 2: 0.70–3.49 kU/L, 3: 3.50–17.49 kU/L, 4: 17.5–49.9 kU/L, 5: 50–99.9 kU/L, 6: > 100 kU/L

On day 8, ventilation showed no signs of improvement despite the treatment, and the patient was still requiring full ECMO support. We administered 600 mg of omalizumab subcutaneously according to the patient’s body weight of 55 kg and the IgE level. We saw the first improvement in ventilation parameters after 90 min. Over the 12 h after omalizumab administration, the patient’s tidal volumes increased from 100 to 500 ml, minute ventilation increased from 1 to 5 L/min, and ECMO gas flow could be stopped. The following day, ECMO was disconnected. A 5-day course of meropenem was started for ventilator-associated pneumonia. Ventilation continued to improve, and the patient was weaned from sedation and mechanical ventilation, and extubated on day 10. Two weeks after the first dose, a second dose of omalizumab was administered. The patient was discharged home on day 25. Table 2 presents the results of pulmonary function tests upon discharge. Her asthma has been well controlled since hospital discharge. Oral prednisone was tapered to discontinuation, and the patient has been treated with a fixed combination of high-dose inhaled beclomethasone/formoterol, oral montelukast and levocetirizine. No additional dose of omalizumab was required.

**Table 2:** Pulmonary function test results upon hospital discharge

	Pre-bronchodilator		Post-bronchodilator	
	Best	%Predicted	Best	%Predicted
FVC (L)	3.85	100.8	3.93	103.1
FEV_1_ (L)	3.05	91.6	3.31	99.5
FEV_1_/FVC	0.79	–	0.84	–
DLCO	–	–	–	90
RV/TLC	–	–	0.43	

*FVC* forced vital capacity, *FEV*_*1*_ forced expiratory volume in the first second, *DLCO* diffusing capacity of the lungs for carbon monoxide, *RV* residual volume, *TLC* total lung capacity

## Discussion

To the best of our knowledge, this is the second published case report of omalizumab treatment in a patient with refractory status asthmaticus.

Omalizumab is a humanized recombinant monoclonal anti-IgE antibody indicated for adults and pediatric patients 6 years of age and older with moderate to severe persistent asthma, a positive skin test or in vitro reactivity to a perennial aeroallergen, and whose symptoms are inadequately controlled by inhaled corticosteroids. It is not currently indicated for the relief of acute bronchospasm or status asthmaticus [7]. However, the decision to administer omalizumab for the unapproved indication of status asthmaticus was supported by several facts. First, the patient had been fully dependent on ECMO for 7 days, with no signs of improvement despite therapy. Second, a case of successful use of omalizumab in a patient with refractory status asthmaticus and a high IgE level has been previously reported [7]. Third, the patient had strong polyvalent atopic sensitization, and there is evidence supporting the efficacy of omalizumab in other IgE-meditated diseases such as chronic spontaneous urticaria, allergic rhinitis, nasal polyposis and food allergy, regardless of IgE level [8]. Cases of successful and safe treatment of pruritic bullous pemphigoid, severe atopic dermatitis and rare hyperimmunoglobulin-IgE syndrome, where the IgE level commonly reaches 2000–5000 IU/L, have also been reported [9], as well as the successful use of omalizumab in cases of bronchial asthma with IgE levels higher than 700 IU/L [10]. Therefore, we believed that the potential benefit of omalizumab outweighed its possible side effects.

The course of our case was very similar to the previously published case report [7], with a remarkably fast and significant effect of omalizumab on ventilatory status. The first effect on ventilation was seen within hours. Bronchial spasm completely resolved within 12 h after the administration of omalizumab, and the patient’s ventilatory status normalized the following day. The patient was extubated three days later.

## Conclusion

The presented case and the previously published report show that omalizumab was very effective in treating refractory status asthmaticus and the administration of omalizumab changed the seemingly unfavorable outcome of these patients. No side effects were noted. Therefore, we believe that omalizumab should be considered in status asthmaticus patients who have high IgE levels and are refractory to standard treatment. Although severe cases of acute asthma exacerbation unresponsive to standard therapy are rare, a future small clinical trial of omalizumab in an acute setting should be considered.

## Data Availability

Data are available upon request.
